# Surveillance on A/H5N1 virus in domestic poultry and wild birds in Egypt

**DOI:** 10.1186/1743-422X-10-203

**Published:** 2013-06-22

**Authors:** Elham F El-Zoghby, Mona M Aly, Soad A Nasef, Mohamed K Hassan, Abdel-Satar Arafa, Abdullah A Selim, Shereen G Kholousy, Walid H Kilany, Marwa Safwat, E M Abdelwhab, Hafez M Hafez

**Affiliations:** 1 National Laboratory for Veterinary Quality Control on Poultry Production, Animal Health Research Institute, Dokki, P.O. Box 246, Giza 12618, Egypt; 2 Institute of Poultry Diseases, Free University of Berlin, Koenigsweg 63-14163, Berlin, Germany; 3 Current address: Federal Research Institute for Animal Health, Friedrich Loeffler Institute – Institute of Molecular Biology, Suedufer 10, Greifswald Insel-Riems 17493, Germany

**Keywords:** Egypt, Highly Pathogenic Avian Influenza H5N1, Epidemiology, Surveillance, Commercial Farms, Backyard Birds, Live Bird Markets, Wild Birds

## Abstract

**Background:**

The endemic H5N1 high pathogenicity avian influenza virus (A/H5N1) in poultry in Egypt continues to cause heavy losses in poultry and poses a significant threat to human health.

**Methods:**

Here we describe results of A/H5N1 surveillance in domestic poultry in 2009 and wild birds in 2009–2010. Tracheal and cloacal swabs were collected from domestic poultry from 22024 commercial farms, 1435 backyards and 944 live bird markets (LBMs) as well as from 1297 wild birds representing 28 different types of migratory birds. Viral RNA was extracted from a mix of tracheal and cloacal swabs media. Matrix gene of avian influenza type A virus was detected using specific real-time reverse-transcription polymerase chain reaction (RT-qPCR) and positive samples were tested by RT-qPCR for simultaneous detection of the H5 and N1 genes.

**Results:**

In this surveillance, A/H5N1 was detected from 0.1% (n = 23/) of examined commercial poultry farms, 10.5% (n = 151) of backyard birds and 11.4% (n = 108) of LBMs but no wild bird tested positive for A/H5N1. The virus was detected from domestic poultry year-round with higher incidence in the warmer months of summer and spring particularly in backyard birds. Outbreaks were recorded mostly in Lower Egypt where 95.7% (n = 22), 68.9% (n = 104) and 52.8% (n = 57) of positive commercial farms, backyards and LBMs were detected, respectively. Higher prevalence (56%, n = 85) was reported in backyards that had mixed chickens and waterfowl together in the same vicinity and LBMs that had waterfowl (76%, n = 82).

**Conclusion:**

Our findings indicated broad circulation of the endemic A/H5N1 among poultry in 2009 in Egypt. In addition, the epidemiology of A/H5N1 has changed over time with outbreaks occurring in the warmer months of the year. Backyard waterfowl may play a role as a reservoir and/or source of A/H5N1 particularly in LBMs. The virus has been established in poultry in the Nile Delta where major metropolitan areas, dense human population and poultry stocks are concentrated. Continuous surveillance, tracing the source of live birds in the markets and integration of multifaceted strategies and global collaboration are needed to control the spread of the virus in Egypt.

## Background

The unprecedented spread of H5N1 high pathogenicity avian influenza virus (A/H5N1) from Asia to Africa in 2005 was considered as a global epidemiological twist [1] due to poor infrastructure of poultry industry and diagnostic laboratory and lack of “accredited” preparedness control plans. Infection of domestic poultry with A/H5N1 in Egypt since mid-February 2006 caused enormous losses in poultry industry and the slaughter-campaign has overwhelmed the resources of the Egyptian veterinary and public health authorities [2]. Thereafter, Egypt has adopted a strategy to combat the disease based mainly on mass vaccination of backyard birds by inactivated H5N1 vaccine provided by the government free of charge (no longer supplied) whereas the commercial sector applied vaccination programmes with widely varying standards. Several types of inactivated H5N1 and H5N2 vaccines with different seed viruses were supplied by a number of vaccine manufacturers and used in the field [2,3].

The capacity of the commercial poultry sector in Egypt was estimated to be 850 million birds in 2006, where the majority of farms are small-scale units (5000 – 20000 birds) with poor or no biosecurity and usually used for broiler and layer poultry production. Conversely, the breeders and grandparent farms have strict biosecurity measures with all-in all-out production systems. Backyard birds in Egypt are a major source for cheap animal protein and essential financial resources for the farmers and small enterprises in rural areas. The backyard sector estimated to have 250 million chickens, ducks, geese, turkeys and rarely pigeons which are usually kept together in the same house [2]. In addition, due to insufficient slaughterhouses, marketing facilities and cultural preference for consumption of freshly slaughtered poultry trading of poultry meat in Egypt depends mainly on live bird markets (LBMs) [4]. In LBMs, birds of different species with various ages from several locations and different sources (backyards/barnyards and commercial flocks) are usually mixed. Therefore, LBMs are an indicator for A/H5N1 infections in poultry. Previous surveillance in Egypt has highlighted continuous and wide circulation of the virus in vaccinated and non-vaccinated commercial farms, backyard birds and LBMs [3-10] and bird-to-human transmission has occurred due to contact and/or slaughtering and defeathering of infected backyard birds [11]. Genetic analysis indicated that the Egyptian A/H5N1 has diversified into multiple genotypes where at least two distinct genotypes are currently prevalent: the 2.2.1.1 clade isolated mainly from vaccinated commercial poultry (and rarely from backyard birds) and the 2.2.1/C viruses isolated from backyard birds, small-scale commercial poultry and human [12,13].

**Figure 1:**
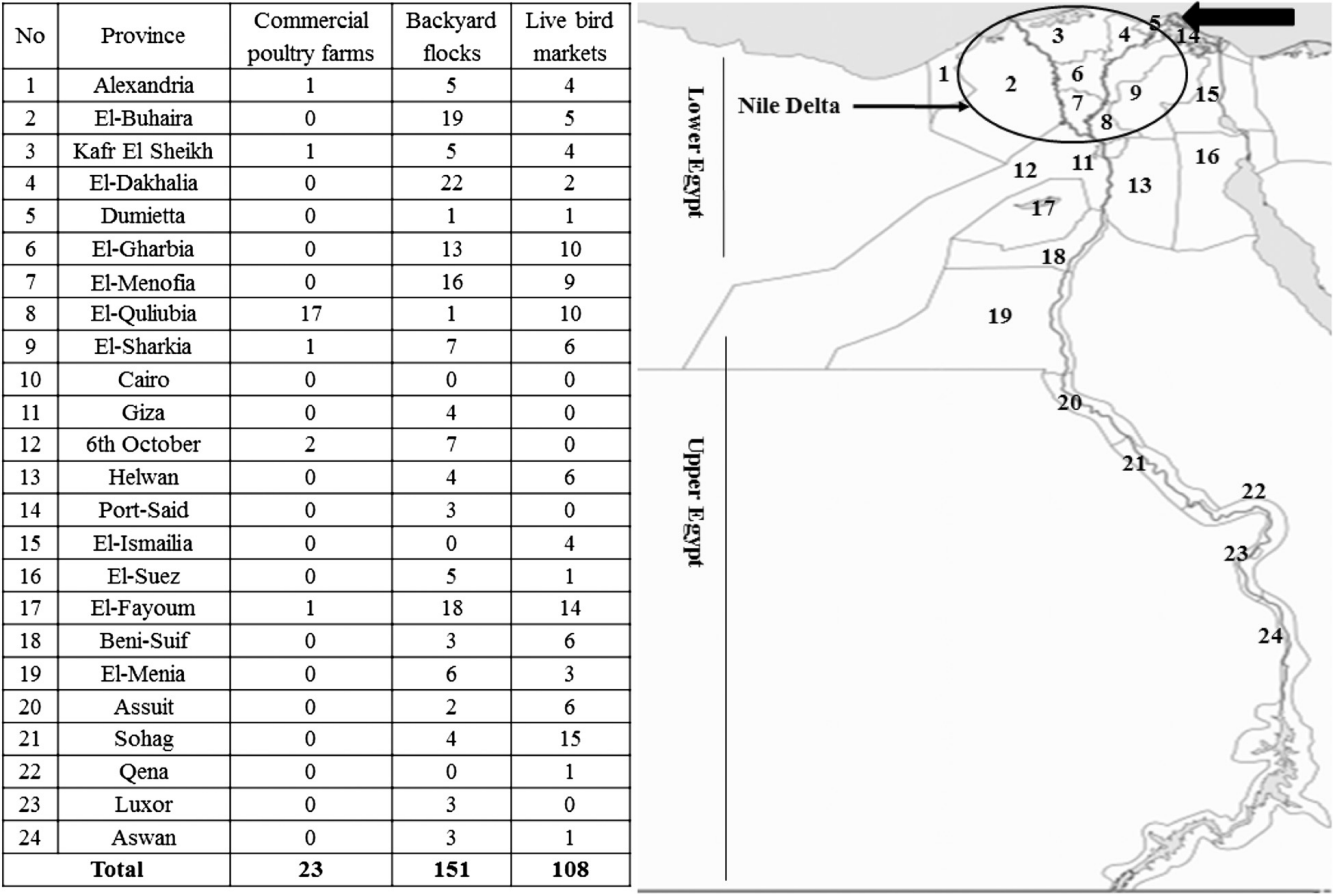
**Geographical distribution of avian influenza H5N1 outbreaks in commercial farms, backyards and LBMs in Egypt in 2009.** Provinces numbers 1 to 13 are located in Lower Egypt, numbers 14 to 16 in the Canal region and numbers 17 to 24 in Upper Egypt. Provinces numbers 1 to 9 is forming the Nile Delta region. Black arrow refers to the location of El-Manzala Lake where four provinces share borders “provinces 4, 5, 9 and 14”.

On the other hand, Egypt acts as a bridge between Europe, Asia and Africa and millions of migrating birds pass through Egypt on their flights annually particularly in winter seasons where the northern Nile Delta lakes act as a major refuge for a multitude of bird species. Lake El-Manzala in the Nile Delta (north-east of Egypt: 32.20 East, 31.27 North) is one of the largest wetland on the Egyptian Mediterranean Coast (about 77,000 ha) where four provinces share its borders (Figure 1). It is an important wetland for wild birds either migratory or winter visitors along the Black-Sea-Mediterranean migratory flyway [14]. Many low pathogenic avian influenza viruses have been isolated from several species of migratory birds in Egypt [15-18] and A/H5N1 virus was possibly introduced into domestic poultry in 2006 by an infected common teal duck near Lake El-Manzala [18].

In previous publications we described the surveillance conducted in domestic poultry in 2006 [3], and 2007–2008 [7]. Here we describe the results of our nationwide surveillance on A/H5N1 in commercial poultry farms, backyard birds and LBMs in Egypt in 2009 as well as in wild birds in Lake El-Manzala in 2009–2010.

## Results

### Field investigations

Clinical signs and lesions of birds in the surveillance was varied; from clinically healthy birds to cyanosis of comb (snout in turkey) and wattle, haemorrhages on the shank, respiratory and intestinal disorders. In layer and breeder flocks complete cessation to slight decrease in egg production was recorded. Congestions and haemorrhages, particularly in parenchymatous organs, were observed. All wild birds tested in this study were apparently healthy.

### Results of virus detection by RT-qPCR

In this surveillance samples were obtained from poultry on 22024 commercial farms, 1435 backyard flocks and 944 LBMs from Lower and Upper Egypt in 2009. The detection rate of A/H5N1 was 0.1% (n = 23/22024) of the examined commercial poultry farms as shown in Table 1. There were 1, 2, 8 and 10 infected broiler breeder, layer breeder, layer and broiler farms, respectively and 2 infected duck farms recorded in 2009 (Table 2). Moreover, 60% (n = 14/23) and 8.7% (n = 2/23) of positive commercial poultry farms used H5N1 and H5N2 vaccines, respectively (Table 2). The virus was detected in commercial farms in each season where 3, 7, 7 and 6 infected farms were detected in winter, spring, summer and autumn, respectively (Table 1).

**Table 1:** Seasonal distribution of A/H5N1 in commercial poultry farms, backyard birds and live bird markets in Egypt during 2009

**Season**	**Commercial farms**		**Backyard flocks**		**Live bird markets**	
	**Examined**	**Positive**	**Examined**	**Positive**	**Examined**	**Positive**
Winter	3665	3 (0.08%)	481	51 (10.6%)	252	36 (14.3%)
Spring	6086	7 (0.12%)	403	48 (11.9%)	400	47 (11.75%)
Summer	6710	7 (0.10%)	163	25 (15.3%)	245	22 (9%)
Autumn	5563	6 (0.11%)	388	27 (6.9%)	47	3 (6.4%)
Total	22024	23 (0.1%)	1435	151 (10.5%)	944	108 (11.4%)

**Table 2:** Type of birds and vaccines used in positive commercial poultry farms infected with A/H5N1 in Egypt in 2009

**Type of birds/vaccine used**	**Number of outbreaks in commercial chicken farms**				**Number of outbreaks in commercial duck farms**	**Total**
	**Broiler breeders**	**Layer breeders**	**Layers**	**Broilers**		
H5N1	0	0	7	7	0	14 (60%)*
H5N2	1	0	0	0	1	2 (8.7%)
Unknown	0	2	1	2	1	6 (26%)
Unvaccinated	0	0	0	1	0	1 (4.3%)
Total	1	2	8	10	2	23 (100%)

* Percent refers to number of infected farms /total of 23 positive farms.

**Figure 2:**
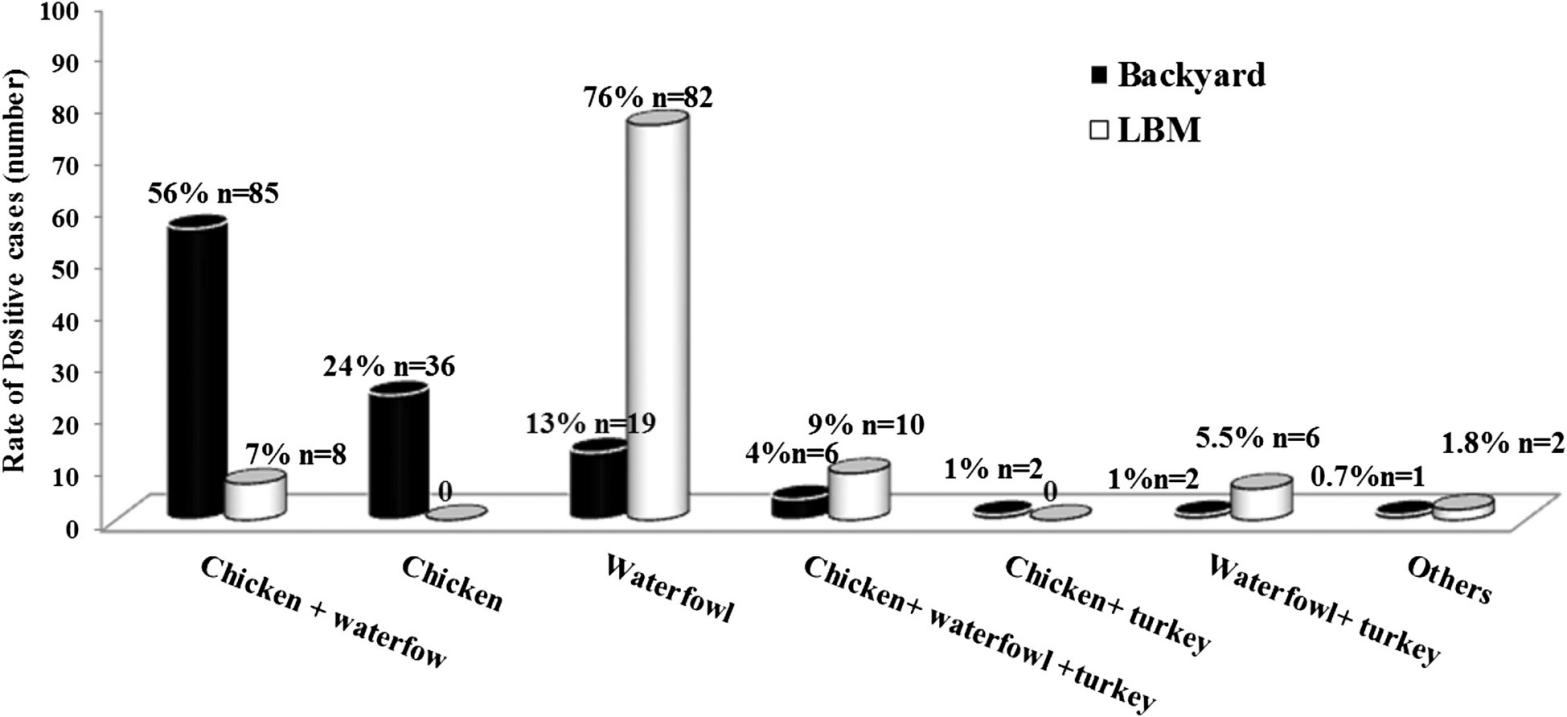
**Poultry infection with A/H5N1 in backyards and live bird markets (LBM) in Egypt in 2009 detected by RT-qPCR.** Species present in the examined backyards and LBMs. Percent refer to positive backyards or LBMs per species/total 151 positive backyards or 108 positive LBMs, respectively. Waterfowl represent duck and/or geese. Others in backyard refer to sample was taken from letter whereas in LBM refers to LBM sell only turkeys.

On the other hand, a total of 10.5% (n = 151/1435) of the examined backyard flocks were detected positive (Table 1). Incidence of the virus was higher in summer (p < 0.05) and spring in backyards where 15.3% (n = 25/163) and 11.9% (n = 48/403) of examined backyard flocks were positive, respectively in comparison to 10.6% (n = 51/481) in winter and 6.9% (n = 27/388) in autumn. Prevalence of A/H5N1 subtype virus in different backyard birds were 56% (n = 85/151), 24% (n = 36/151) or 13% (n = 19/151) of positive backyards had chicken-waterfowl, chicken or waterfowl, respectively (Figure 2).

Results of our surveillance revealed that 11.4% (n = 108/944) of the LBMs were positive for A/H5N1 (Table 1). The detection rate of A/H5N1 in winter, spring, summer and autumn was 14.3% (n = 36/252), 11.8% (n = 47/400), 9% (n = 22/245) and 6.4% (n = 3/47), respectively. The highest prevalence of H5N1 was in LBMs which had waterfowl (76%; n = 82/108), while LBMs that had waterfowl-chicken-turkey; waterfowl-chicken and waterfowl-turkey represented 9% (n = 10/108), 7% (n = 8/108) and 5.5% (n = 6/108), respectively (Figure 2). The detection rate of A/H5N1 in commercial poultry was significantly lower than that in backyards and LBMs (p <0.05) whereas the detection rate in LBMs was not significantly different than that in backyards (p = 0.39). There was a medium positive correlation (r = 0.31) between seasonal incidence of the virus in poultry in backyards and LBMs whereas seasonal incidence of the virus in commercial poultry was negatively correlated with incidences in backyards and LBMs (r = - 0.1 and - 0.52, respectively).

Out of 1297 examined wild bird samples only one teal duck was found positive for the matrix gene of AIV but not for H5 and/or N1 genes (Table 3). Attempts to isolate this virus were unsuccessful (data not shown).

**Table 3:** Number of samples collected from different wild birds in Lake El-Manzala

**Number of collected swabs**	**Type of wild bird**
331	Coot
195	Teal duck
166	Cormorant
120	Quail
95	Shoveler
60	Purple swamp-hen
58	Moorhen gallinula
53	Stock dove
41	Pintail
32	Great egret
25	Common moorhen
19	Ferruginous duck/ Mahogany/ White-eyed pochard
16	Little crake
16	Squacco heron
12	Mallard/wild or Mammon duck
10	African sacred ibis
10	Egyptian vulture
10	Wigeon duck
8	Rose-ringed parakeet
5	Slender billed gull
4	Kestrel
2	Black headed gull
2	Dalmatian pelica
2	Gerfalcon
2	Tufted duck
1	Common pochard
1	House sparrow
1	Lanner falcon
1297	Total

### Spatial distribution of the virus

There were 95.7% (n = 22/239) positive commercial farms in Lower Egypt and only one farm in Upper Egypt. In addition, there were 68.9% (n = 104/151) positive backyard flocks in Lower Egypt and 25.8% (n = 39/151) in Upper Egypt while 5.3% (n = 8/151) were reported in the Canal region (Figure 1). Moreover, the incidence of the disease in LBMs was higher in Lower Egypt as 52.8% (n = 57/108) of surveyed markets were positive in comparison to 42.6% (n = 46/108) in Upper Egypt and 4.6% (n = 5/108) in the Canal region. Respectively, 87% (n = 20/23), 59% (n = 89/151) and 47% (n = 51/108) of positive commercial farms, backyards and LBMs were reported in the Nile Delta as shown in Figure (1).

### Sequence and phylogenetic analyses

**Figure 3:**
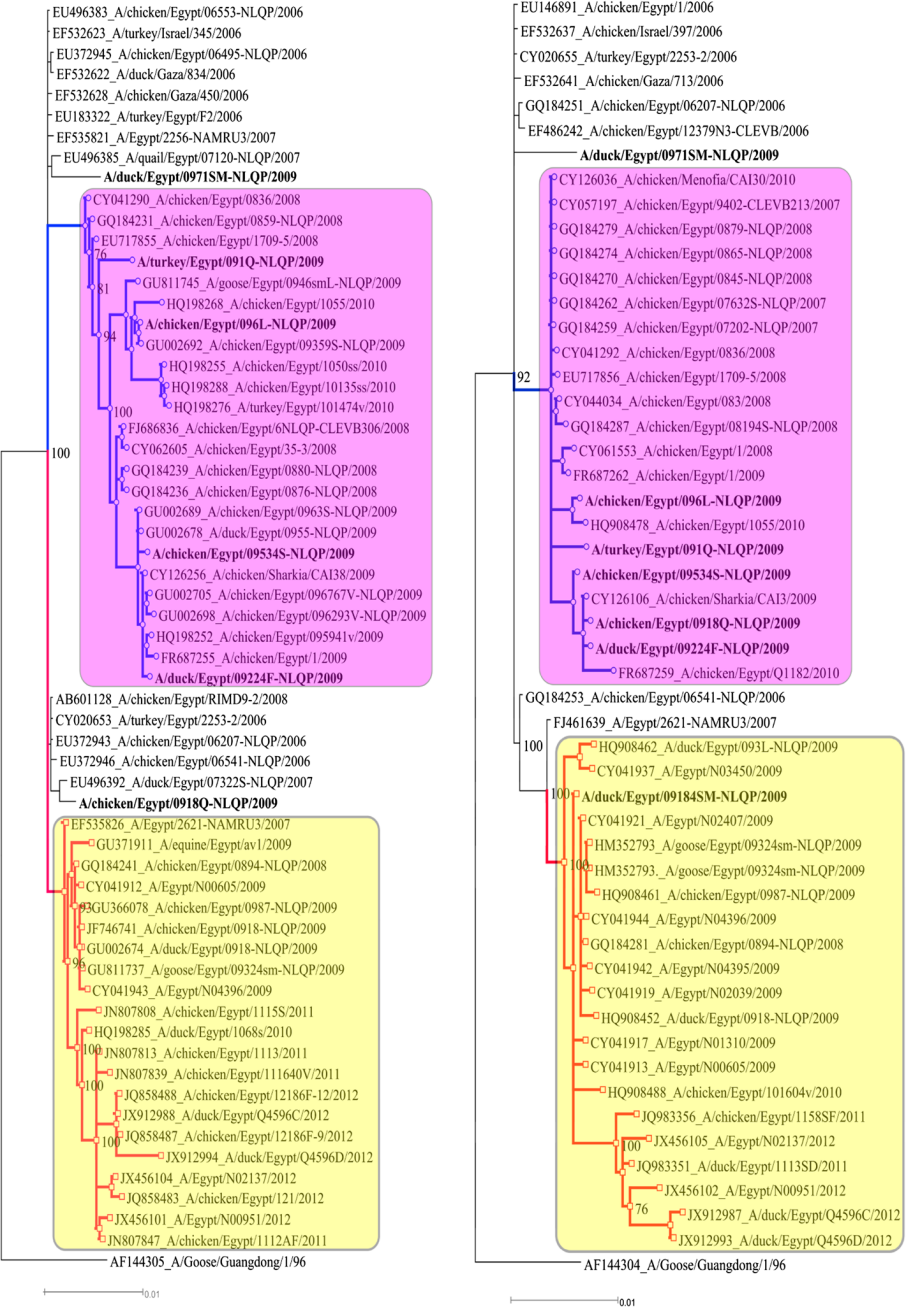
**Phylogenetic relatedness of the HA and NA genes generated in this study with other relevant H5N1 viruses isolated from poultry and human in Egypt.** Phylogenetic relatedness of the H5 (left) and N1 (right) sequences generated in this study and relevant genes were done using the Bayesian (MrBayes algorithm) method launched from TOPALi v2 with general time-reversible (GTR) likelihood model and 10 independent runs with the initial 25% of each run discarded as burn-in. Each run consisted of two independent tree files with each 100,000 Markov Chain Monte Carlo-sampled trees. Viruses obtained from this study are written in bold and the two co-circulating genotypes are highlighted in pink (variant clade 2.2.1.1) and yellow (2.2.1/c subclade). Both trees were rooted to the corresponding sequences of A/goose/Guangdong/1/96. Final trees were further edited by Inkscape 0.48 software.

Sequence of the HA and/or NA genes of seven randomly selected viruses from chickens, ducks or turkeys in different poultry sectors from Upper and Lower Egypt was generated (Table 4). The topology of the phylogenetic trees was similar in all methods (data not shown). As shown in Figure 3, the phylogenetic analysis of the HA gene indicated that four viruses (Ck6-BY, Tk1-M, Ck534-BY and Dk224-F) are located within the 2.2.1.1 clade characterized by mutations in residues 74, 97, 110, 123, 140, 141, 144, 154, 156, 162, 165, 184, 226 and 238 (Figure S1) and shared 97.1 – 97.5% and 95.4 – 96.3% nucleotides and amino acids identity with the parent virus, respectively (Additional file 1: Table S1). Whereas two viruses (Dk71-M and Ck18-F) clustered within two different extinct groups [12] and shared more than 98% identity with the parent virus. Except for the Ck18-F (isolated from poultry in a commercial farm in El-Qaluibia province) which clustered within the variant 2.2.1.1 clade characterized by T168I substitution (Additional file 2: Figure S2), the topology of NA was similar to the HA and shared 98.2 – 98.5% and 99.1 – 99.7% nucleotide and amino acid identity with the parent virus, respectively (Additional file 3: Table S2). The NA sequence of Dk184-BY virus isolated from backyard ducks clustered with the classic 2.2.1/C group characterized by A46D, L204M, S319F and S430G substitutions (Additional file 2: Figure S2) and respectively shared 98.9 and 99.3% nucleotide and amino acid identity with the parent virus (Additional file 3: Table S2).

**Table 4:** Viruses subjected to sequence analysis in this study and their GenBank accession numbers

**No.**	**Virus**	**Abbreviation**	HA*	NA*	**Sector**	**Province**	**Date of isolation**
1	A/duck/Egypt/0971SM-NLQP/2009	Dk71-M	GU002697	HQ908471	LBM	El-Monofia	February-2009
2	A/chicken/Egypt/0918Q-NLQP/2009	Ck18-F	GU002687	HQ908466	Farm	El-Qaluibia	March-2009
3	A/chicken/Egypt/096 L-NLQP/2009	Ck6-BY	GU811726	HQ908463	Backyard	Luxor	May-2009
4	A/turkey/Egypt/091Q-NLQP/2009	Tk1-M	GU002702	HQ908465	LBM**	El-Sharkia	January-2009
5	A/chicken/Egypt/09534S-NLQP/2009	Ck534-BY	GU002694	HQ908470	Backyard	6th October	May-2009
6	A/duck/Egypt/09224 F-NLQP/2009	Dk224-F	GU002686	HQ908464	Farm	El-Qaluibia	May-2009
7	A/duck/Egypt/09184SM-NLQP/2009	Dk184-BY	Not done	HQ908472	Backyard	Assuit	March-2009

* accession numbers, **LBM = live bird market.

## Discussion

Active surveillance on avian influenza virus in Egypt has been extensively performed on a regular basis since February 2006. For instance, our surveillance in 2009 confirmed continuous A/H5N1 infections in commercial poultry farms, backyard birds and LBMs in Egypt. Although, outbreaks in commercial poultry farms (0.1%, n = 23/22024) could be neglected as a great risk for the commercial poultry sectors in contrast to the backyards (10.5%, n = 151/1435). However, lack of compensation hampers the reporting of outbreaks and might result in a skewed/blurred picture of the actual field situation. It is worth mentioning that the current capacity of slaughterhouses in Egypt was estimated roughly to be 30 – 60% of the national meat poultry production and veterinary inspection is insufficient [2]. Moreover, illegal trading of unexamined commercial poultry and backyard birds into LBMs is not uncommon; therefore this might explain the higher incidence of the virus in LBMs (11.4%, n = 108/944). These results are in accordance with our surveillance conducted in cooperation with the Food and Agriculture Organization of the United Nations (FAO) in 2006 [3] and 2007 – 2008 [7]. In contrast to our results, Kayali et al. [19] reported 6.8% (n = 192/2827) positivity rate in commercial farms, 3.3% (n = 34/1024) in LBMs and only 0.9% (n = 12/1381) in the backyard flocks. However, the latter group conducted a targeted surveillance from August 2009 to July 2010 in six governorates only where 53% (n = 2959/5562) of their collected samples were from poultry from two governorates.

The viral circulation in vaccinated and non vaccinated birds was previously reported; particularly during the winter seasons of 2006 – 2008 [3,7]. However, the results obtained herein showed that the epidemiology of A/H5N1 in Egypt in 2009 has changed over time with outbreaks, especially in backyards, occurred in the warmer months of the year; spring and summer which may indicate establishment and adaptation of the virus to the environmental conditions. This observation is in accordance with findings of Kayali et al. [19] in Egypt in 2009–2010 and was also found in Vietnam [20] and in opposition to a winter-associated pattern of AIV in other countries [21-23]. In the current study, A/H5N1 was more prevalent in LBMs that had waterfowls (and/or turkeys) but not chickens alone. Unfortunately, paucity of epidemiological data is an obstacle for identification of the source of birds, particularly waterfowl, in the markets and curbs trace back of infection. Nevertheless, due to cultural factors the source of ducks in LBMs is usually the backyards while chickens are usually come from commercial farms [24]. Also, in contrast to chickens, waterfowl can be silently infected with A/H5N1 [25-29] which may maintain the virus in the LBMs for longer periods. It has been previously described that A/H5N1 infections are high in Upper Egypt particularly in the Nile Delta which could be considered as the influenza epicentre in Egypt where major metropolitan areas with dense human populations are concentrated and a lot of poultry are likely to be traded and consumed [3,7,10,13,19,26].

Previous comprehensive phylogenetic analyses described temporal pattern of A/H5N1 in Egypt and neither geographical nor species-linked pattern were observed [12,13]. Interestingly, Ck18-F and Dk224-F were isolated from two different poultry farms at the same governorate (El-Qaluibia) but they belonged to two different genetic lineages (Figure 3) whereas Ck6-BY and Tk1-M isolated from LBM and commercial farms, respectively from two different provinces (about 800 km apart) clustered together within the 2.2.1.1 clade. This could be explained by the rapid and random movement of poultry nationwide and mix of different poultry and marketing sectors. The topology of the HA gene of Ck18-F isolated from commercial poultry is different from the topology of the NA gene (Figure 3) which possibly is due to reassortment. However, the full genome sequence is required to confirm this notion.

Although isolation of many AIV subtypes from wild birds in Egypt has been previously reported, we failed to identify any A/H5N1 from wild birds which may indicate, in the context of this surveillance, no role of wild birds for spread of the virus in domesticated poultry. This is also in accordance with previous negative A/H5N1 results in samples collected from wild birds in Lake El-Manzala in 2006 [30] and 2009 [16]. Indeed, commercial poultry-LBMs-backyards cycle in Egypt is closely integrated and any breach will eminently affect poultry and endanger public health. Therefore we suggest that enforcement of biosecurity measures should be the first line of defence while vaccination acts only as an ancillary tool for control of A/H5N1. Depopulation of infected holdings requires prompt and fair compensation. Lack thereof will severely hamper effective eradication of the disease.

## Conclusion

Our findings indicated broad circulation of the endemic A/H5N1 among poultry in 2009 in Egypt. In addition, the epidemiology of A/H5N1 has changed over time with outbreaks occurring in the warmer months of the year. Backyard waterfowl may play a role as a reservoir and/or source of A/H5N1 particularly in LBMs. Continuous surveillance, tracing the source of live birds in the markets and integration of multifaceted strategies and global collaboration are needed to control the disease in poultry in Egypt.

## Materials and methods

### Surveillance

Samples were collected during the routine nationwide avian influenza surveillance program after the ministerial decree number 221/2006 in charge the National Laboratory for Veterinary Quality Control on Poultry Production (NLQP) for official diagnosis and surveillance for avian influenza virus (AIV) in Egypt. The surveillance in domestic poultry was conducted in 2009 in 24 out of 29 provinces in Egypt which allocated in Lower Egypt (13 provinces), Upper Egypt (8 provinces) and Canal region (3 provinces) as shown in Figure (1). Nile Delta (9 provinces) is located in Lower Egypt between Damietta and the Rosetta branches of river Nile and represents approximately 4% of Egypt area where 95% of human population and poultry are living together. Surveillance was carried out in commercial poultry farms, backyard birds and LBMs. Samples were collected from some commercial farms on more than one occasion whereas each backyard and LBM was visited only once. In this study, a commercial farm was considered as an epidemiological unit regardless of the number of houses or flocks in the farm. Likewise, the house was considered as an epidemiological unit regardless of the type of backyard birds or species. Therefore, the number of positive backyard holdings is the number of positive backyard flocks. Also, the positive LBMs refer to the market rather than the species.

Up to 20 cloacal and tracheal swabs were collected from 22024 commercial poultry farms, 1435 backyards and 944 LBMs (Table 1). A maximum of ten tracheal or cloacal swabs collected from commercial poultry were pooled separately and the examined sample was a mix of tracheal and cloacal swabs. Swabs from each species in each of surveyed backyards or LBMs were pooled together; however for economical reasons examined samples in the laboratory represent the house or the LBM as a whole; if multiple species were present. The available history of vaccination of poultry in positive farms is summarized in Table 2.

The surveillance in wild birds was conducted during January 2009 and January, September and October 2010 in Lake El-Manzala. Wild birds were captured by hands or using mist nets, traps or shot by professional hunters. Cloacal and/or tracheal swabs were collected from 1297 wild birds representing 28 different types where 907 (69.9%) samples were obtained from Coot, Teal, Cormorant, Quail and Shoveler (Table 3). All swab samples were collected on viral transport medium containing antibiotics, transported to NLQP without breaking the cold chain and then stored at -80°C until used [31].

### Real-time reverse-transcription polymerase chain reaction (RT-qPCR)

RNA was extracted from a mix of cloacal and tracheal swabs by using a MagNA Pure LC Total Nucleic Acid Extraction kit according to the manufacturer’s instructions using a MagNA Pure LC instrument (Roche, Mannheim, Germany). Samples were amplified using One-step Real Time RT-PCR Kit (Qiagen, Valencia, Calif.) for detection of type A avian influenza viruses targeting the matrix gene using primers and probe described by Spackman et al. [32]; forward primer 5′-AGA TGA GTC TTC TAA CCG AGG TCG-3′, reverse primer 5′-TGC AAA AAC ATC TTC AAG TCT CTG-3′and probe 5′ FAM-TCA GGC CCC CTC AAA GCC GA-TAMRA-3′. The test was conducted in a Stratagene MX3005P real time PCR machine as mentioned before [3]. Positive AIV samples were used for further H5N1 subtype identification using H5N1 Real Time RT-PCR Kit (Roche Diagnostics Ltd) following the instructions of the manufacturer using LightCycler® 2.0 machine (Roche, Mannheim, Germany).

### Sequence and phylogenetic analyses

Seven viruses were randomly selected from chickens (n = 3 viruses), ducks (n = 3) and turkeys (n = 1) from commercial farms (n = 2), backyards (n = 3) and LBMs (n = 2) in Lower (n = 5) and Upper Egypt (n = 2) as shown in Table 4. The nucleotide sequence of a total of 6 HA and 7 NA genes was generated as previously described [5] using BigDye Terminator v3.1 Cycle Sequencing Kit on an automatic sequencer (ABI-3130; Applied Biosystems, Foster City, CA). The obtained sequences were assembled and aligned to the related A/H5N1 viruses using MAFFT [33], BioEdit version 7.0.9.0 [34] and the results were further enhanced by manual editing. The generated sequences were deposited in the GenBank database and their accession numbers are listed in Table 4. Nucleotide and amino acid identity matrices of sequences generated from this study and the putative parent virus (A/chicken/Egypt/06207-NLQP/2006) isolated during the first outbreak in February 2006 were calculated. Amino acids of H5 and N1 proteins were numbered according to the mature protein of the putative parent virus. Phylogenetic relatedness of the H5 and N1 sequences conducted in this study and relevant genes retrieved from the GenBank were done using the Bayesian (MrBayes algorithm) method launched from TOPALi v2 [35] with general time-reversible (GTR) likelihood model and 10 independent runs with the initial 25% of each run discarded as burn-in. Each run consisted of two independent tree files with each 100,000 Markov Chain Monte Carlo-sampled trees. The resultant trees were compared with the consensus trees of 1000 bootstrap replicates produced by neighbor-joining, maximum-likelihood and maximum-parsimony implemented in MEGA5 [36]. All trees were rooted to the corresponding sequences of A/goose/Guangdong/1/96. Final trees were further edited by Inkscape 0.48 software (Figure 3).

### Statistics

Chi-square and Pearson product moment correlation coefficient were used to analyse difference and correlation of A/H5N1 infections in poultry sectors in different seasons.

### Availability of supporting data

The data sets supporting the results of this article are included within the article (and its additional files; Additional file 4: Figure S1, Additional file 2: Figure S2, Additional file 1: Table S1 and Additional file 3: Table S2.

## Abbreviations

A/H5N1: H5N1 high pathogenicity avian influenza virus; AIV: Avian influenza viruses; FAO: Food and agriculture organization of the united nations; HA: Hemagglutinin; NA: Neuraminidase; LBMs: Live bird markets; NLQP: National laboratory for veterinary quality control on poultry production; OIE: World organization for animal health; RT-qPCR: Real-time reverse transcription polymerase chain reaction.

## Competing interests

The authors declare that they have no competing interests.

## Authors’ contributions

EFE, AS, SGK, WHK, MS, carried out sample collection and examination. AA carried out the sequence of the selected viruses. EMA did sequence and phylogenetic analyses and prepared the manuscript, MMA, SN, MKH and HMH conceived and coordinated the study and revised the manuscript. All authors read and approved the final manuscript.

## Supplementary Material

Additional file 1: Table S1 Sequence identity matrix of the HA of viral sequences generated in this study compared to corresponding sequence of the putative parent virus (^nucleotides^ identical _amino acids_). Click here for file

Additional file 2: Figure S2 Alignment of amino acid sequences of the NA protein generated compared to the corresponding sequence of the putative parent virus (A/chicken/Egypt/06207-NLQP/2006). Click here for file

Additional file 3: Table S2 Sequence identity matrix of the NA of viral sequences generated in this study compared to corresponding sequence of the putative parent virus (^nucleotides^ identical _amino acids_). Click here for file

Additional file 4: Figure S1 Alignment of amino acid sequences of the HA protein generated compared to the corresponding sequence of the putative parent virus (A/chicken/Egypt/06207-NLQP/2006). Click here for file
