# Supplementary material for: Surveillance on A/H5N1 virus in domestic poultry and wild birds in Egypt
Source: Virol J. 2013 Jun 22;10:203. doi: 10.1186/1743-422X-10-203 (PMC3699397; doi:10.1186/1743-422X-10-203)
Supplement: Additional file 1: Table S1 — Sequence identity matrix of the HA of viral sequences generated in this study compared to corresponding sequence of the putative parent virus (nucleotides identical amino acids). [file 1743-422X-10-203-S1.doc]

**Table S1.** Sequence identity matrix of the HA of viral sequences generated in this study compared to corresponding sequence of the putative parent virus (^nucleotides^ **identical** _amino acids_)

| No. | Virus | Abbreviation | Genotype | 1 | 2 | 3 | 4 | 5 | 6 | 7 |
| --- | --- | --- | --- | --- | --- | --- | --- | --- | --- | --- |
| 1 | EU372943_A/chicken/Egypt/06207-NLQP/2006 | Parent virus | 2.2.1 | **ID** | 99.2 | 98.3 | 97.2 | 97.1 | 97.5 | 97.5 |
| 2 | A/chicken/Egypt/0918Q-NLQP/2009 | Ck18-F | Extinct | 99.0 | **ID** | 97.6 | 97.4 | 97.3 | 97.4 | 97.5 |
| 3 | A/duck/Egypt/0971SM-NLQP/2009 | Dk71-M | Extinct | 98.1 | 97.2 | **ID** | 95.9 | 95.8 | 96.3 | 96.1 |
| 4 | A/duck/Egypt/09224F-NLQP/2009 | Dk224-F | 2.2.1.1 | 96.0 | 96.5 | 95.1 | **ID** | 99.4 | 98.2 | 97.8 |
| 5 | A/chicken/Egypt/09534S-NLQP/2009 | Ck534-BY | 2.2.1.1 | 95.8 | 96.3 | 94.7 | 99.6 | **ID** | 98.1 | 97.8 |
| 6 | A/chicken/Egypt/096L-NLQP/2009 | Ck6-BY | 2.2.1.1 | 96.3 | 96.5 | 95.6 | 97.8 | 97.4 | **ID** | 98.0 |
| 7 | A/turkey/Egypt/091Q-NLQP/2009 | Tk1-M | 2.2.1.1 | 95.4 | 96.0 | 94.7 | 96.9 | 96.5 | 96.7 | **ID** |
