# Supplementary material for: Surveillance on A/H5N1 virus in domestic poultry and wild birds in Egypt
Source: Virol J. 2013 Jun 22;10:203. doi: 10.1186/1743-422X-10-203 (PMC3699397; doi:10.1186/1743-422X-10-203)
Supplement: Additional file 2: Figure S2 — Alignment of amino acid sequences of the NA protein generated compared to the corresponding sequence of the putative parent virus (A/chicken/Egypt/06207-NLQP/2006). [file 1743-422X-10-203-S2.doc]

**Figure S2.** Alignment of amino acid sequences of the NA protein generated compared to the corresponding sequence of the putative parent virus (A/chicken/Egypt/06207-NLQP/2006)

**
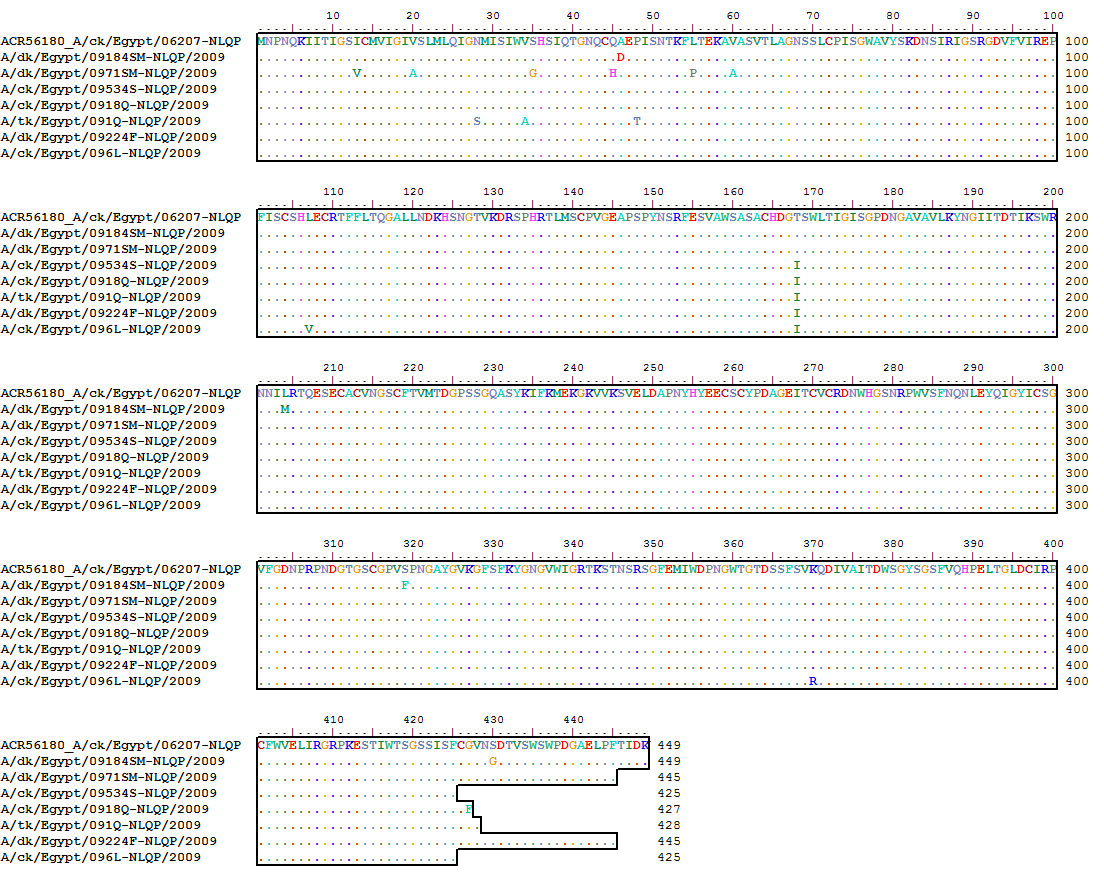
**
